# Proliferative verrucous leukoplakia: diagnosis, management and current advances^☆^

**DOI:** 10.1016/j.bjorl.2016.12.005

**Published:** 2017-01-24

**Authors:** Diogo Lenzi Capella, Jussara Maria Gonçalves, Adelino António Artur Abrantes, Liliane Janete Grando, Filipe Ivan Daniel

**Affiliations:** a Universidade Federal de Santa Catarina (UFSC), Programa de Pós-graduação em Odontologia, Florianopolis, SC, Brazil; b Universidade Federal de Santa Catarina (UFSC), Departamento de Patologia, Florianopolis, SC, Brazil

**Keywords:** Leukoplakia oral, Leukoplakia, Proliferative verrucous leukoplakia, Oral cancer, Squamous cell carcinoma, Head and neck cancer, Leucoplasia oral, Leucoplasia, Leucoplasia verrucosa proliferativa, Câncer oral, Carcinoma, Célula escamosa, Câncer de cabeça e pescoço

## Abstract

**Introduction:**

Proliferative verrucous leukoplakia is a multifocal and progressive lesion of the oral mucosa, with unknown etiology, and commonly resistant to all therapy attempts with frequent recurrences. It is characterized by a high rate of oral squamous cell carcinoma and verrucou carcinoma transformations.

**Objective:**

To analyze the studies about Proliferative verrucous leukoplakia and develop a concise update.

**Methods:**

A Pubmed search identifying studies (laboratory research, case series and reviews of literature) that examined patients with Proliferative verrucous leukoplakia was realized.

**Results:**

There are not enough studies about Proliferative verrucous leukoplakia in the literature. The few found studies not present a consensus about its etiology and diagnosis criteria. Although several treatment strategies have been proposed, most of them still show a high recurrence rate.

**Conclusion:**

More research about Proliferative verrucous leukoplakia is necessary to understand and treat this disease.

## Resumo

**Introdução:**

Leucoplasia verrucosa proliferativa (LVP) é uma lesão multifocal e progressiva da mucosa oral, com etiologia desconhecida e comumente resistente a todas as tentativas terapêuticas, com recorrências frequentes. É caracterizada por uma alta taxa de transformação em carcinoma de células escamosas e carcinoma verrucoso da cavidade oral.

**Objetivo:**

Analisar os estudos sobre LVP e elaborar uma atualização resumida.

**Método:**

Foi realizada uma pesquisa na base de dados Pubmed identificando estudos (pesquisas laboratoriais, séries de casos e revisões de literatura) que avaliaram pacientes com LVP.

**Resultados e discussão:**

Não há estudos suficientes sobre LVP na literatura. Os poucos estudos encontrados não apresentam consenso quanto aos critérios de etiologia e diagnóstico. Embora várias estratégias de tratamento tenham sido propostas, a maioria ainda apresenta alta taxa de recorrência.

**Conclusão:**

Mais pesquisas sobre LVP são necessárias para entender e tratar esta doença.

## Introduction

Proliferative verrucous leukoplakia (PVL) is a very aggressive and rare form of oral leukoplakia (OL) with high morbidity.^1^ The first description has been made by Hansen et al. (1985) as a distinct form of OL which develops initially as a white plaque that eventually becomes multifocal slow-growing lesions resistant to all therapeutic procedures, including surgery, with a high recurrence rate and an oral cancer transformation trend.^2^ With the introduction of the term PVL, the previously used term “oral florid papillomatosis” has disappeared from the literature.^3^ Actually, the World Health Organization (WHO) (2005) described PVL as “a rare but distinctive high-risk clinical form of oral precursor lesions”.^4^ Several studies have examined PVL characteristics and its propensity to develop into oral carcinoma.^4^ Thirty years after its discovery, it is still a challenging disease with no confirmed etiology and efficient treatment. Although there are published papers about PVL diagnosis criteria, they may be imprecise in detecting early disease presentations, either for clinical or histopathological view. The objective of this paper is to analyze the PVL literature and to develop a concise update.

## Review methods

A PubMed search using the term “Proliferative Verrucous Leukoplakia” was made from 1985 to 2015 (30 years). Additional papers were included based upon the original literature search and references in the selected papers. Papers concerning laboratory research, case series, as well as reviews of literature were also included.

## Results and discussion

### Etiology

Hansen et al. (1985) described PVL as a disease with unclear etiology, but typically associated with tobacco use.^2^ However, the role of tobacco in PVL lesions is unknown since these lesions are seen in smokers and nonsmokers (Table 1).^1^, ^2^, ^5^, ^6^, ^7^, ^8^, ^9^, ^10^, ^11^, ^12^, ^13^, ^14^, ^15^ Several studies evaluated alcohol use by PVL patients, but the relation between them was not stablished (Table 1).^1^, ^9^, ^11^, ^13^

**Table 1:** Studies of PVL cases series.

Authors	No. of cases	Age Mean (range)	Sex (M/F)	Tobacco use	Alcohol use	Follow-up (years, mean)	Malignant transf.		Recurrence
Hansen et al. (1985)^2^	30	65.9 (27–90)	6/24	18	Non-reported	6.1^a^^,^^b^^,^^d^	VC	9	90%
							OSCC	17	
Kahn et al. (1994)^15^	4	72.25 (75–79)	2/2	2	0	4^a^^,^^c^	VC	1	75%
							OSSC	2	
Zakrzewska et al. (1996)^5^	10	63.6 (42–81)	5/5	7	Non-reported	7.5^a^^,^^b^^,^^d^	VC	4	90%
							OSCC	6	
Silverman and Gorsky (1997)^6^	54	62 (22–89)	11/43	17	Non-reported	11.6^a^^,^^b^^,^^d^	OSCC	38	85%
Fettig et al. (2000)^7^	10	65.2 (51–82)	6/4	3	Non-reported	5^b^^,^^c^	VC	3	100%
							OSCC	5	
Bagan et al. (2003)^8^	30	70.97 (84–58)	6/24	7	Non-reported	4.7^b^^,^^c^	VC	8	86.7%
							OSCC	19	
Ghazali et al. (2003)^9^	9	61.6 (24–76)	2/7	4	1	4.7^b^^,^^c^	VC	–	55.5%
							OSCC	–	
Campisi et al. (2004)^1^	58	66.5 (54–79)	22/36	17	10	Non-reported	VC	3	Non-reported
							OSCC	22	
Bagan et al. (2007)^10^	13	68.3 (45–86)	0/13	3	Non-reported	Non-reported	OSCC	6	Non-reported
							VC	0	
Klanrit et al. (2007)^11^	6	65.8 (56–81)	1/5	1	1	6^c^	–	3	Non-reported
Morton et al. (2007)^12^	3	80 (73–89)	1/2	1	Non-reported	3.7^c^	VC	1	66.6%
							OSCC	2	
Gandolfo et al. (2009)^13^	47	65.9 (40–86)	10/37	17	12	6.89^b^^,^^c^	VC	9	Non-reported
							OSCC	32	
Bagan et al. (2011)^14^	55	61.69 (73–50)	19/36	20	Non-reported	7.53^b^^,^^c^	OSCC	27	85%
Gouvêa et al. (2013)^21^	21	65.5 (79–52)	3/18	Non-reported	Non-reported	7.38^b^^,^^c^	OSCC	7	Non-reported
							VC	2	

VC, verrucous carcinoma; OSCC, oral squamous cell carcinoma.

a Follow up until the cure.

b Follow up during proliferative verrucous leukoplakia progress.

c Follow up until malignant transformation.

d Follow up until death.

In recent years, it has been hypothesized that human papillomavirus (HPV) may influence both potentially and already stablished oral malignant lesions.^16^ Although the association between oral squamous cell carcinoma (OSCC) and HPV is already mentioned, its influence on PVL cases is not confirmed yet.^17^ Over the last decades, some studies reported different and contradictory frequencies of HPV DNA detection in PVL (Table 2).^1^, ^7^, ^10^, ^18^, ^19^

**Table 2:** Studies about HPV presence in PVL.

Author	No. of cases	HPV positive	HPV types
Palefsky et al. (1995)^18^	9	8 (88.8%)	HPV 16 (*n* = 7; 77.7%)
			HPV 18 (*n* = 1; 11.1%)
Gopalakrishnan et al. (1997)^19^	10	2 (20%)	HPV 16 (*n* = 1; 10%)
			HPV 18 (*n* = 1; 10%)
Fettig et al. (2000)^7^	10	0	–
Campisi et al. (2004)^1^	58	14 (24.1%)	HPV18 (*n* = 10; 17.24%)
			HPV 16 (*n* = 4; 6.8%)
Bagan et al. (2007)^10^	13	0	–

HPV, human papillomavirus; PVL, proliferative verrucous leukoplakia.

About other possible etiologies, there are few studies with PVL that tried to identify the presence of *Candida albicans*. Silverman et al. (1997) reported 19 of 38 specimens with *C. albicans* positivity, but without correlation between the fungal infection and PVL occurrence or progression to carcinoma, characterizing it as a probable secondary infection.^6^ Similarly, Hansen et al. (1985) observed that 12 of 30 patients were positive for *C. albicans*.^2^ Concerning to Epstein Barr Virus (EBV), Bagan et al. (2008) was the only one to detect EBV in a PVL group (60% of 6 patients).^20^ Therefore, none of these studies have yet established the exact role of microbiological agents in PVL pathogenesis.

### Epidemiological and clinical characteristics

In the studies evaluated in this paper, PVL occurred predominantly in women, with a 2.72:1 (female/male) rate, and a mean age of 66.8 years (Table 1).^1^, ^2^, ^5^, ^6^, ^7^, ^8^, ^9^, ^10^, ^11^, ^12^, ^13^, ^14^, ^15^, ^21^ The most affected sites were gingiva,^8^, ^9^, ^10^, ^12^, ^14^, ^15^ buccal mucosa,^2^, ^5^, ^6^ and alveolar ridge,^11^, ^13^ while the tongue was less involved.^21^

**Figure 1:**
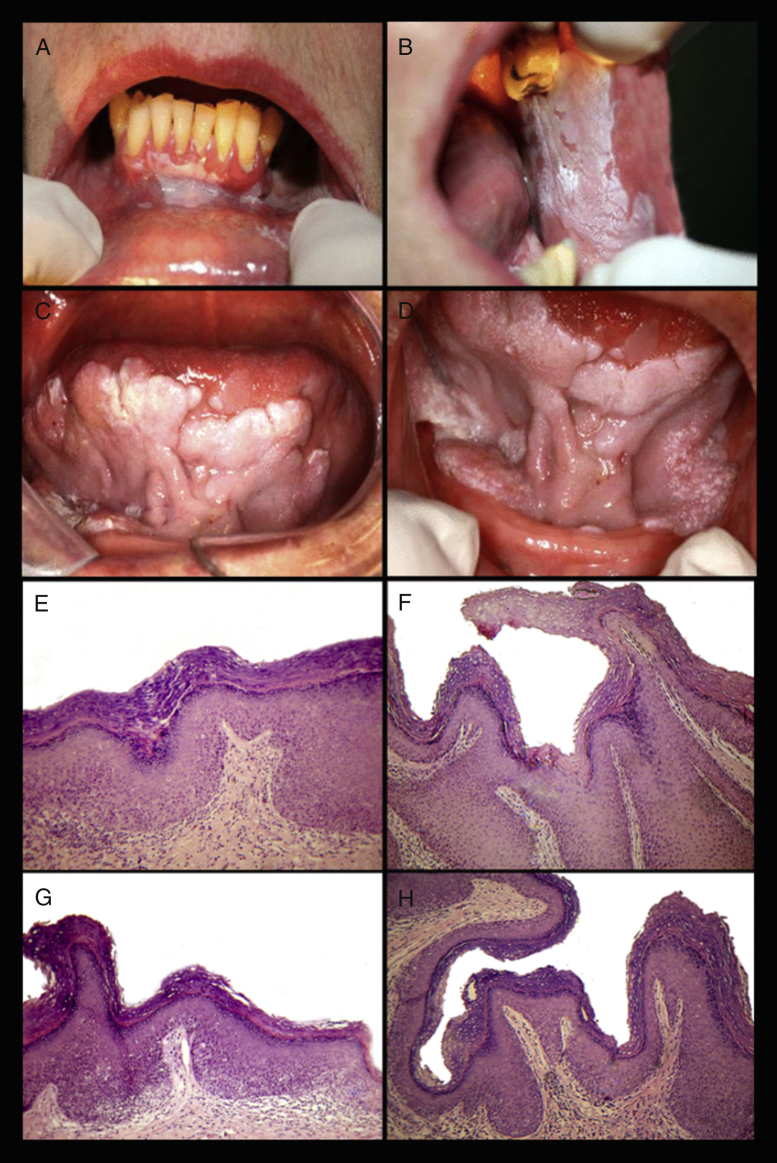
(A) Proliferative verrucous leukoplakia (PVL) in the lower attached gingival, vestibular sulcus and gradually extended along left alveolar ridge. (B) PVL in the buccal mucosa with different clinical patterns: larger areas of homogeneous leukoplakias and spot areas of thickening of the keratinization and/verrucous surface. (C and D) PVL in ventral tongue and floor of mouth with exophytic appearance and focal area of granular pattern in both alveolar ridges. (E) Histopathological view showing acanthosis and hyperkeratosis with mild dyplasia. (F) Exophytic, hyperkeratotic lesion with prominent verruciform or papillary surface and acanthosis forming blunt projections into the lamina propria. (G) Hyperkeratosis, acanthosis, irregularity of the basal layer and some areas of epithelial atrophy. (H) Hyperkeratosis with droplet-shaped epithelial projections and intact lamina propria (HE, original magnification 40×).

Zakrzewska et al. (1996) observed that initial PVL clinical features included small whitish and well-defined signs of non-homogeneous leukoplakic lesions with speckled pattern.^5^ According to Ghazali et al. (2003), PVL initially presents as unifocal, homogeneous, slow and persistent growth lesion.^9^ At this stage, it is extremely difficult, if not impossible, to distinguish it from oral leukoplakia. PVL has one or more areas of homogeneous leukoplakia, which grows slowly and persistently, and tends to become multifocal with exophytic, verrucous, or erythematous areas.^22^ After a long period, commonly six years, the evolution to carcinoma occurs.^23^ Areas that are erythematous, verrucous or have papillary surface are characteristic of malignant transformation, and so these areas should have a histopathological confirmation (Fig. 1).^23^

Recently, a PVL subtype designated proliferative verrucous leukoplakia of the gingiva (PVLG) has been reported as involving exclusively free and attached gingiva. PVLG is characterized as a whitish plaque, unifocal, recurrent and progressive lesion. The course is also unpredictable and may undergo OSCC or verrucous carcinoma (VC) transformation.^7^

As the evolution stages of different sites in multifocal lesions are not necessarily the same, patients should be monitored closely, with frequent and repetitive biopsies when there are changes in color, appearance or size, and when new lesions appear.^9^, ^24^, ^25^ Patients with whitish harmless appearance and recurrence episodes should also be followed up every six months.^25^ PVL may progress to VC or OSCC over time in spite of numerous treatment interventions, suggesting that PVL is associated with diffuse submicroscopic changes of the oral mucosa, sometimes described as “field cancerization”.^24^ Therefore, PVL presents a high malignant transformation rate (Table 1).^26^

### Histopathology

Histopathological findings may show acanthosis and hyperkeratosis with an interface lymphocytic infiltrate within the superficial lamina propria. If the lesions continue to grow horizontally and vertically, there are histopathological changes that increase roughness of surface with verrucous aspect, and hyperplasia with or without dysplasia.^12^ Therefore, over time and without treatment, there is an inexorable progression to VC or OSCC (Fig. 1).^5^, ^12^, ^22^, ^27^

**Figure 2:**
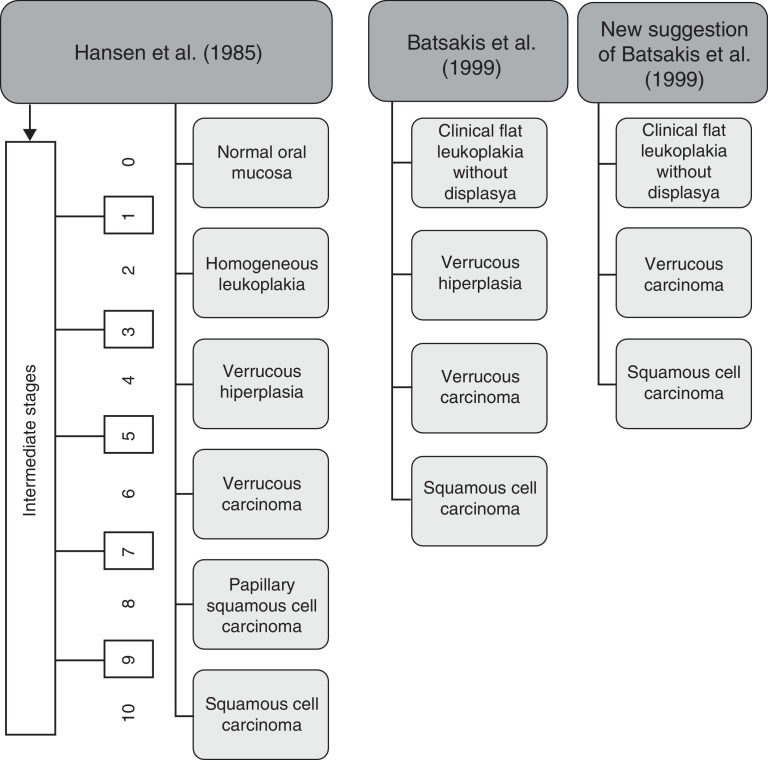
Histologic stages of progression to carcinoma. Adapted from Ghazali et al. (2003).^9^

Hansen et al. (1985) described the histopathological progression of PVL in 10 stages during its clinical course.^2^ This classification divided PVL in five groups: hyperkeratosis, verrucous hyperplasia (VH), VC, papillary squamous cell carcinoma and OSCC poorly differentiated, with intermediate stages. Frequently the lesions move slowly up in grade, with very few reverting cases. However, Murrah and Batsakis (1999) reduced the number of histologic stages to four, removing the intermediate stages, and proposed a review that omitted papillary squamous cell carcinoma considering it a PVL independent entity more frequent in the oropharynx.^28^ Batsakis et al. (1999) also considered the possible removal of VH since it has much in common with VC, but with an exophytic growth pattern in VH in opposition to an endophytic growth pattern in VC (Fig. 2).^29^

### Biomarkers

Recent findings have indicated that carcinogenesis is a result of accumulated genetic and epigenetic alterations that may lead to chromosomal instability, in the form of numerical or structural aberrations, which might be detected as abnormal DNA content or aneuploidy.^30^ Ploidy analysis in PVL was performed in three different studies with a high prevalence. Khan et al. (1994) analyzed 4 PVL by flow cytometry and found DNA aneuploid in all cases.^15^ Klanrit et al. (2007) analyzed 6 paraffin-embedded PVL samples and detected 4 (66.6%) cases with abnormal ploidy status prior to malignant transformation.^11^ Gouvea et al. (2013) analyzed DNA of 20 patients with PVL and 19 (95%) cases showed aneuploidy, with abnormal DNA observed even in the more indolent lesions.^21^

Therefore, several studies have been conducted to determine whether improved expression levels of some molecular markers involved in different cellular pathways can be valuable indicators of clinical behavior.^31^ Gopalakrishnan et al. (1997) studied p53 expression in 10 samples and found minimal markup in normal oral mucosa, but positivity in 8 (80%) of the cases of PVL, and in 7 (70%) of the cases of OSCC.^19^ Fettig et al. (2000) identified p53 expression in 4 (40%) of the 10 analyzed cases.^7^ Gouvea et al. (2013) showed p53 immunoreactivity in 14 (77.7%) of the 18 cases.^32^ In the same study Ki-67 expression was similar to the one observed with p53 protein progression of epithelial dysplasia in PVL. Ki-67 is a nuclear protein associated with cellular proliferation with potential predictive biomarker in early stages of OSCC and can be used in addition to conventional tumor staging for optimal therapeutic management.^33^ Akrish et al. (2015) realized a retrospective review of 11 patients with PVL, 38 with carcinoma arising in patients with PVL (P-SCC) and 49 with conventional squamous cell carcinoma (C-SCC).^34^ Overexpression of p53 was more common in P-SCC, but without ki-67 or p16 overexpression. Krest et al. (2014) evaluated cell cycle regulatory genes in 20 PVL cases and detected deletion or mutation event involving both p16INK4a and p14ARF genes in 45% of the cases.^35^

### Diagnostic criteria

Hansen et al. (1985) determined that lesions diagnosed as PVL could have initially a homogeneous aspect, without dysplasia, followed by warty appearance of surface areas and multiple discrete or confluent lesions in single or multiple intra-oral sites.^2^

According to Cerero-Lapiedra et al. (2010), studies published on PVL followed the diagnostic criterion postulated by Hansen et al. (1985).^2^, ^23^ It is a pioneering and comprehensive description, but still needs updates. Therefore, the same authors proposed the reformulation of the criteria by dividing them into major (five criteria) and minor (four criteria) (Table 3). To PVL diagnosis, patient should have one of the following combinations:

- Three major criteria (one of which must include the evolution of the histopathological lesions).
- Two major criteria (one of which must include the evolution of the histopathological lesions) + two minor criteria.

**Table 3:** Proposal of major and minor diagnostic criteria for PVL recommended by Cerero-Lapiedra et al. (2010).^23^ Adapted from Carrard et al.^36^

Major criteria		Minor criteria	
A	Leukoplakia lesion with more than two different oral sites. It is frequently found in the gingiva, alveolar processes and palate.	A	Oral leukoplakia lesion that occupies at least 3 cm when adding all the affected areas.
B	The existence of a verrucous area.	B	Female patient
C	Lesions that spread or engross during the disease development	C	Non-smoker patient (male or female).
D	There has been a recurrence in a previously treated area.	D	More than 5 years of evolution.
E	Can vary from simple epithelial hyperkeratosis to OSCC, whether in situ or infiltrating.	–	–

PVL, proliferative verrucous leukoplakia.

Bagan et al. (2011) believed that these criteria are useful only for those with clinical experience with PVL, but can be confusing for beginners.^14^ Corroborating with this observation, Carrard et al. (2013) suggested simplifying the diagnostic criteria by omitting the distinction between major and minor criteria. However, all four criteria should be met (Table 4).^36^

**Table 4:** Modified diagnostic criteria for PVL recommended by Carrard et al. (2013).^36^ Adapted from Carrard et al.^36^

1. Leukoplakia showing the presence of verrucous or wartlike areas, involving more than two oral subsites.
2. When adding all involved sites the minimum size should be at least three centimeters.
3. Well documented period of disease evolution of at least five years, characterized by spreading and enlarging and the occurrence of one or more recurrences in a previously treated area.
4. The realization of at least one biopsy (to rule out the presence of a VC or OSCC).

PVL, proliferative verrucous leukoplakia.

### Treatment and recurrence

This literature review (Table 5) showed different treatments modalities. Surgery and laser ablation were the most used. Ten papers utilized surgery in 136 cases^2^, ^5^, ^6^, ^7^, ^8^, ^9^, ^11^, ^12^, ^14^, ^15^ and seven papers used laser ablation in 64 cases.^2^, ^5^, ^7^, ^8^, ^11^, ^14^, ^15^ According to evaluated studies, with at least 30 patients, we found a recurrence mean rate of 85% for all treatment modalities isolated or associated.^1^, ^2^, ^6^, ^8^, ^13^, ^14^

**Table 5:** Treatments applied in cases series from literature.

Treatment	Author	Number of cases	Total
Radiation	Hansen et al. (1985)^2^	18	18
Chemotherapy	Hansen et al. (1985)^2^	6	6
Surgery	Hansen et al. (1985)^2^	22	136
	Kann et al. (1994)^15^	2	
	Zakrzewska et al. (1996)^5^	1	
	Silverman and Gorsky (1997)^6^	42	
	Bagan et al. (2003)^8^	24	
	Ghazali et al. (2003)^9^	8	
	Klanrit et al. (2007)^11^	6	
	Morton et al. (2007)^12^	2	
	Bagan et al. (2011)^14^	21	
	Fettig et al. (2000)^7^	8	
Surgery and radiation	Hansen et al. (1985)^2^	11	23
	Zakrzewska et al. (1996)^5^	1	
	Silverman and Gorsky (1997)^6^	11	
Surgery and ablation laser (CO_2_)	Hansen et al. (1985)^2^	1	2
	Zakrzewska et al. (1996)^5^	1	
Ablation laser (CO_2_)	Hansen et al. (1985)^2^	2	64
	Kahn et al. (1994)^15^	2	
	Zakrzewska et al. (1996)^5^	2	
	Bagan et al. (2003)^8^	5	
	Fettig et al. (2000)^7^	18	
	Klanrit et al. (2007)^11^	1	
	Bagan et al. (2011)^14^	34	
Photodynamic therapy and laser ablation	Zakrzewska et al. (1996)^5^	4	4
Block resection	Fettig et al. (2000)^7^	1	1
Retinoid	Poveda-Roda et al. (2010)^37^	16	18
	Hansen^2^ (1985)	2	

There are 2 descriptions about the association between surgery and laser ablation with no improvement.^2^, ^5^ Zakrzewska et al. (1996) showed one patient with no recurrence at the laser-treated sites, but new lesions developed elsewhere.^5^ Bagan et al. (2003), after treating 24 patients (80%) with surgery and 18 (60%) with laser ablation, detected recurrence rate of 86.7% and recognized new lesions in 83.3%.^8^ Fettig et al. (2000) identified that both simple excision and laser excision were ineffective in eradicating lesions.^7^ Surgery, despite high recurrence rates, gives the possibility of dysplasia histologic grading and early detection of malignant transformation. Laser ablation should be indicated for lesions where the surgery would be contraindicated by lesion size or access difficulty. Development of new lesions in these patients is constant; thus, multiple interventions are always necessary.

Hansen et al. (1985) utilized radiation in 16 patients and chemotherapy in 6, with only one patient free of PVL at 6 years after treatment; therefore, they concluded that radiation therapy is not entirely satisfactory in a widespread disease such as PVL.^2^ In spite of these results, others papers reported the association between radiation and surgery to treat PVL, totaling 24 cases described in the literature.^2^, ^5^, ^6^ Silverman et al. (1997) reported that radiation was not effective in controlling PVL based on the lack of response of the cases treated with radiotherapy.^6^ Zakrzewska et al. (1996) treated one patient with radiotherapy, but lesions continued to appear throughout the mouth.^5^ One patient also received a limited course of chemotherapy, but new lesions appeared, demonstrating the ineffectiveness of these treatment. Radiotherapy or chemotherapy did not show improvement in lesion recurrence, and showed severe side effects such as mucositis, infection, and salivary gland problems.

Extensive surgery such as resection was performed in only one case by Fettig et al. (2000).^7^ According to the authors, local block resection was required to prevent recurrences. In spite of this report, one case is not sufficient to confirm the potential of this therapy modality. In addition to its radical and debilitating characteristic, extensive resection is only acceptable when OSCC transformation with bone invasion is present.

Photodynamic therapy (PDT) associated to laser ablation would appear to offer slight improvement prognosis, because it makes treatment of multifocal areas possible with acceptable morbidity, but it did not prevent new lesions and until the moment there is only one study demonstrating its efficacy.^5^

A preliminary study of Poveda-Roda et al. (2010) revealed that topical or systemic retinoic acid produces improvement in about one-third of all patients with PVL, but clinical worsening was recorded in another third of cases.^37^ Besides, further studies are needed to assess the safety of these products, because frequent adverse effects can occur. The most frequent adverse effects were cheilitis, desquamation, pruritus, alopecia and rhinitis, which coincided with the well-known retinoid side effects. However, two of the patients suffered serious problems not described in the Summary of Product Characteristics of the medication used; they developed intense rectal bleeding and cramps of the extremities that made standing and walking difficult. Suppression of the drug led to resolution of these manifestations.

## Conclusion

Although there are not enough studies to determine PVL etiology and no simplified diagnosis criteria, the most difficult point is PVL treatment. According to the literature reviewed, PVL seems to be resistant to many therapy attempts and often has high propensity for dysplasia and/or malignancy progression. Modalities such as surgery, laser ablation, photodynamic therapy, retinoid, radiation and chemotherapy are not effective in reducing relapses and malignant transformation.

## Conflicts of interest

The authors declare no conflicts of interest.
